# Efficient Segmentation of a Breast in B-Mode Ultrasound Tomography Using Three-Dimensional GrabCut (GC3D)

**DOI:** 10.3390/s17081827

**Published:** 2017-08-08

**Authors:** Shaode Yu, Shibin Wu, Ling Zhuang, Xinhua Wei, Mark Sak, Duric Neb, Jiani Hu, Yaoqin Xie

**Affiliations:** 1 Shenzhen Institutes of Advanced Technology, Chinese Academy of Sciences, Shenzhen 518055, China; sd.yu@siat.ac.cn (S.Y.); sb.wu@siat.ac.cn (S.W.); 2 Shenzhen College of Advanced Technology, University of Chinese Academy of Sciences, Shenzhen 518055, China; 3 Department of Oncology, the Karmanos Cancer Institute, Wayne State University, Detroit, MI 48201, USA; zhuangling@gmail.com (L.Z.); sakm@karmanos.org (M.S.); duric@karmanos.org (D.N.); 4 Department of Radiology, Guangzhou first Hospital, Guangzhou Medical University, Guangzhou 510180, China; weixinhua@aliyun.com; 5 Delphinus Medical Technologies, Inc., Plymouth, Detroit, MI 46701, USA; 6 Department of Radiology, Wayne State University, Detroit, MI 48201, USA

**Keywords:** ultrasound tomography, whole breast imaging, image segmentation

## Abstract

As an emerging modality for whole breast imaging, ultrasound tomography (UST), has been adopted for diagnostic purposes. Efficient segmentation of an entire breast in UST images plays an important role in quantitative tissue analysis and cancer diagnosis, while major existing methods suffer from considerable time consumption and intensive user interaction. This paper explores three-dimensional GrabCut (GC3D) for breast isolation in thirty reflection (B-mode) UST volumetric images. The algorithm can be conveniently initialized by localizing points to form a polygon, which covers the potential breast region. Moreover, two other variations of GrabCut and an active contour method were compared. Algorithm performance was evaluated from volume overlap ratios (TO, target overlap; MO, mean overlap; FP, false positive; FN, false negative) and time consumption. Experimental results indicate that GC3D considerably reduced the work load and achieved good performance (TO = 0.84; MO = 0.91; FP = 0.006; FN = 0.16) within an average of 1.2 min per volume. Furthermore, GC3D is not only user friendly, but also robust to various inputs, suggesting its great potential to facilitate clinical applications during whole-breast UST imaging. In the near future, the implemented GC3D can be easily automated to tackle B-mode UST volumetric images acquired from the updated imaging system.

## 1. Introduction

More than 1.3 million women worldwide are diagnosed with breast cancer each year, making it the second most common cancer [1,2]. In developed countries, one in eight women might develop this disease in their lifetime [1]; and in underdeveloped countries, the health burden of breast cancer is increasing [2]. It is crucial to screen breast cancer at an earlier stage, since early diagnosis of breast cancer increases treatment options and significantly reduces mortality [3].

As an emerging modality for whole breast imaging, ultrasound tomography (UST) offers many advantages in the screening and diagnosis of breast cancer over commonly used modalities, such as mammography, hand-held ultrasound, computerized tomography (CT) and magnetic resonance imaging (MRI). Above all, it takes about one minute to scan an average breast using its ring array transducer. From the nipple to the chest wall, UST imaging acquires coronal slices of breast anatomy when the breast is surrounded by a water reservoir. With its contoured curves and recessed lighting, this design reduces human subjectivity and tissue deformation [4,5]. Furthermore, it can simultaneously create three distinct volumes among which the reflection (B-mode) imaging shows tissue structure; the transmission imaging measures changes in sound speed; and the attenuation imaging presents quantitative variance in the sound signal interacting with breast tissue [6,7]. With a universal threshold, breast tissues and tumor regions in UST images are comparably rendered as those in MR images [8]. Moreover, UST aids in tumor differentiation, especially for obscured tumors or tumors located within dense breasts. Importantly, UST images show consistency in breast density estimation as that measured by using mammographic images. This suggests UST may also be used to quantify breast cancer risk factors as well as cancer detection [9,10,11,12,13]. In general, the image acquisition is highly efficient, cost effective and safe, using no ionizing radiation.

Whole breast imaging has highlighted the importance of isolating the breast region from its surrounding water in reflection (B-mode) UST images, since B-mode images present breast anatomy and tissue structure with high in-plane resolution. Intuitively, image segmentation enhances the breast visualization, and accordingly, the observation of suspicious region and clinical diagnosis are easier to perform. Breast segmentation also improves quantitative tissue analysis [9,10,11,12,13] and other follow-up applications [14,15]. It has far reaching consequences in the longitudinal analysis of UST images to facilitate the quantification of breast tissue growth during treatment delivery [16,17,18,19], the characterization of physical breast density using intra-patient alignment of UST and MR images [20], and the rendering of breast tumors by co-registration of B-mode images with transmission, attenuation, MR or mammographic images [21,22]. For instance, facilitated by accurate segmentation of the breast in B-mode UST images, parenchymal changes in women undergoing tamoxifen therapy can be quantified and visualized with deformation field in high precision, up to 0.25 mm [16,17,19]. Therefore, women with or without mass lesions and patients receiving treatment, all can benefit from B-mode UST image segmentation to monitor the tissue growth, to detect suspicions regions, or to quantify the treatment outcome.

**Figure 1:**
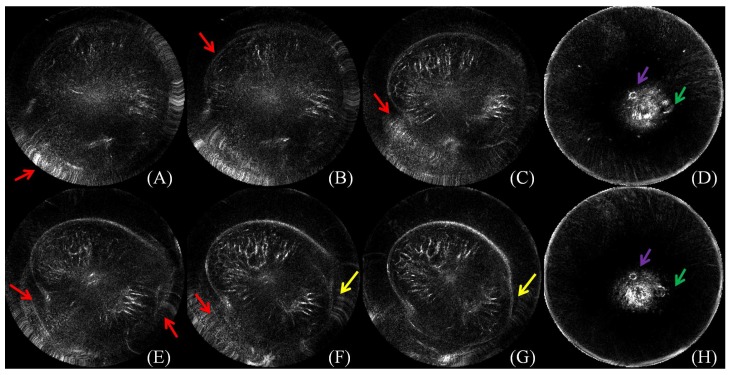
(**A–H**) The challenges of breast segmentation in B-mode ultrasound tomography (UST) images. Red arrows indicate the major artifacts due to the limited space between the breast boundary and the ring transducer; yellow arrows indicate unnatural distortion of the breast shapes; purple arrows indicate the second kind of circle-like artifacts; and green arrows indicate the real nipple regions. Annoying stripes (A–C, E–G) and unnatural breast shapes (C, E–G) are observed. Moreover, artifacts with circle-like shapes (indicated by purple arrows) look like nipples (indicated by green arrows) (D, H). The figure can be enlarged to view the details.

Efficient segmentation of the breast in B-mode UST images is challenging. During the procedure of image acquisition, the breast is immersed in water. Physically, the skin in UST images encloses the breast tissue and the background is approximately zero due to the surrounding water. In an ideal situation, the breast boundary can be distinguishable from its surrounding water region. However, two major kinds of artifacts exist in the clinic. The first one is caused by the limited distance between the chest wall and the ring transducer, which leads to severe distortion when scanning a large-sized breast. The second one is a circle-like shape with various intensities which mainly occurs around the nipple region. Other artifacts come from breathing, particularly from patients who do not have good control of their breathing. Furthermore, in ultrasound imaging, unavoidable noise is considered to be content related [23,24], which further imposes difficulties on B-mode UST image segmentation. Particularly, breasts have various shapes, sizes and densities across patients, and the imaging position is changeable. As shown in Figure 1, artifacts caused by limited space between the chest wall and the ring transducer severely alter image content, causing annoying stripes and unnatural breast shapes. Note that these distorted images should be tackled by manual segmentation. Artifacts may also cause circle-like shapes to look much like nipples, which might cause misleading image interpretation.

To the best of our knowledge, five methods have been used or developed for UST breast image segmentation. Balic et al. [25] used three-dimensional (3D) active contour [26] for breast boundary detection in attenuation UST images. The method is fast and fully automated. However, this method highly depends on the selection of an initial shape and no systematic evaluation has been used to quantify its performance. Hopp et al. [27] integrated edge detection and surface fitting for breast segmentation in reflection images, and experimental validation has verified its feasibility, while more than 40% of slices required manual correction. Sak et al. [9] took advantage of K-means [28] and the thresholding method [29] to handle this problem, while proper parameters were needed in these methods for sound speed images. They also carried out a robustness test on attenuation images, and good to great overlap in the volumes of tissue was achieved [30]. Hopp et al. [31] updated the 3D active contour [32] by applying gradient vector flow [33] and encoded aperture characteristics as additional weighting terms. The algorithm is feasible for automated B-mode images and achieves an average surface deviation from manual segmentation of 2.7 mm [31]. Under this situation, manual segmentation of the breast outline has been widely accepted in ongoing studies [10,11,12,13,14]. Apart from poor time consumption, unstable outcome and intensive user interaction, an additional assumption is imposed in [25,27] that the breast boundary in each coronal slice can be modeled with an ellipse.

Massive algorithms have been proposed for image segmentation [34,35,36,37,38], while few methods are explored to fulfill the necessities of UST image segmentation in terms of accuracy and efficiency. Both the K-means [28] and the thresholding [29] methods are relatively straightforward and only pixel intensities are taken into consideration. Watershed methods classify pixels into regions using gradient descent and analyzes weak points along region boundaries [39,40]. SNAKE (also known as active contour) utilizes splines to fit image structures of lines and edges [26,32,37]. Watershed and SNAKE methods benefit from the distinguishable intensity difference between the breast boundary and its surrounding regions. Notably, for SNAKE, the initialization shape plays an important role in the successful segmentation of the UST images [25]. Whole breast segmentation can also be fulfilled by the graph cuts methods [41,42,43,44,45]. Among these methods, GrabCut [42] is more preferable for B-mode UST images. At first GrabCut integrates texture and edge information, and it models the foreground and the background with Gaussian mixture models (GMMs) [43]. Besides, it substantially simplifies the manual interaction by drawing a rectangle for algorithm initialization that saves time. In addition, GrabCut allows users to incorporate prior knowledge, to validate results and to correct errors in the process of segmentation.

This paper explores the performance of three variations of the GrabCut method in whole breast segmentation using B-mode UST images. One variation is the modified GrabCut (GC) for slice-wise image segmentation and the other two variations are 3D implementations. The first 3D variation takes 6-connected neighboring voxels (GC3D) and the second variation takes 26-connected voxels (GC3D_26) into foreground/background modeling. Additionally, this study compares these results to a previously simplified SNAKE (sSNAKE) [46]. Altogether, the four semi-automated methods are investigated in this paper, and the performance of breast UST image segmentation is evaluated in terms of accuracy, time consumption and ease-of-use.

## 2. Materials and Methods

### 2.1. Data Collection

Thirty reflection (B-mode) UST volumetric images were collected and analyzed (SoftVue${}^{\mathrm{TM}}$, Delphinus Medical Technologies, Plymouth, MI, USA). The reflection imaging reveals the internal anatomical structures of breasts, and therefore, it is much easier to interpret than those images from sound speed imaging and attenuation imaging. The physical resolution of B-mode UST images is [0.5, 0.5, 2.0] mm${}^{3}$, and the matrix size in the coronal plane is [512, 512]. The imaging system, SoftVue${}^{\mathrm{TM}}$, has received FDA clearance for diagnostic imaging purposes and is not intended for use as a replacement for screening mammography (http://www.delphinusmt.com/).

### 2.2. Image Preprocessing

**Figure 2:**
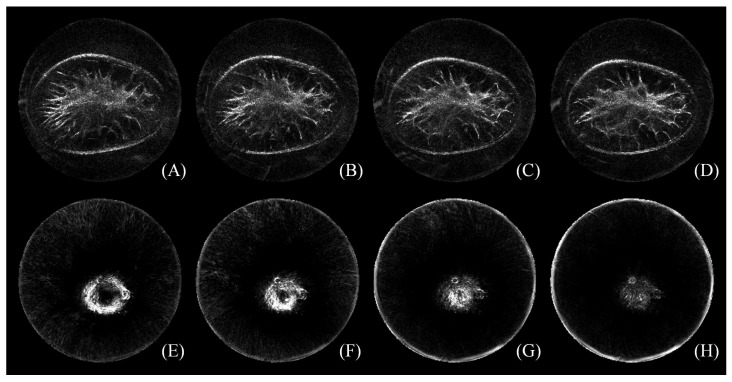
(**A–H**) The process of how to select the starting and the ending slice with a representative image. Under the context of four tomograms in the top row, (B) was chosen to be the first slice because of its visible gap between artifact distortion and the breast boundary; in the bottom row, (G) was chosen as the last slice with the nipple present. The figure can be enlarged to view the details.

A physician with more than fifteen years of work experience was asked to define the starting and the ending slice by using the software package ImageJ [47]. It is observed that slices near the chest wall are distorted by artifacts, while slices around the mammilla could be extensively blurred [13]. How to select the starting and the ending slice is illustrated in Figure 2 with a representative image. The image is composed of 36 slices. The top row shows the 12th–15th tomogram near the chest wall, and the bottom row is the 29th–32nd tomogram near the nipple position. Under the context of selecting the starting and the ending slice, adjacent tomograms should be fully considered. In this case, (B) was chosen to be the first slice because of its visible gap between artifacts and the breast boundary, and (G) was chosen as the last slice with the present nipple. Therefore, the index of remaining slices ranges from the 13th–31st in this case. In other words, 19 slices are left for follow-up image segmentation.

To the dataset in this study, the number of remaining slices ranged from 11 to 21 with an average number of 16 slices. That means 22 mm–42 mm, an average of 32 mm, of the breast was analyzed. In general, 13–18 slices were removed from each image stack as a result of this process.

### 2.3. Benchmark Building

The benchmark (also known as gold standard data) is created by a senior physician with the help of a free-form curve fitting method for slice-wise image processing [46]. The method consists of point localization, Hermite cubic curve interpolation and active contour for boundary segmentation. While different from the original method, the first two steps were employed to outline the breast region, because the points were elaborately located. During the process of placing landmarks by the physician, the number of points can be as many as desired localized on the breast boundary. Then, nine points are uniformly generated between an adjacent point pair using the Hermite cubic curve interpolation [48]. The closed region is masked with the function poly2mask in MATLAB (MathWorks, Natick, MA, USA). After that, outlined regions were further verified by two other physicians to minimize the possible bias. A house-built MATLAB program was implemented to display the original image and its corresponding image with the outlined breast region side by side, and the two physicians worked together to browse the image pair slice by slice. If the breast region was not appropriate, the slice would be re-outlined. Finally, these slice with outlined breast region are stacked to form the ground truth data for the validation of segmentation algorithms.

**Figure 3:**
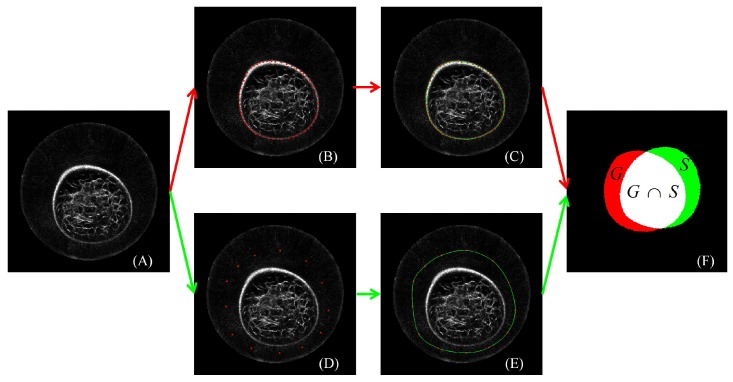
The semantic description of benchmark building, algorithm initialization and performance evaluation with one slice as an example. (**A**) The source image. (**B**) Elaborately manual localization of points on the breast boundary. (**C**) Automated closed curve generation. (**D**) User interaction for algorithm initialization with points placed between the ring transducers and the breast boundary. (**E**) Automated polygon generation. (**F**) Performance quantification by comparing the ground truth *G* and the segmentation result *S*. Note that (A, B, C), (A, D, E) and (F) correspond to the process of benchmark building, algorithm initialization and performance evaluation, respectively. The figure can be enlarged to view the details.

Figure 3 presents the process of the manual outline of the breast region in one slice. A physician first localized points on the breast boundary. Then, the red points in (B) were interpolated by using Hermit curve fitting as shown in (C) with the green line. Finally, the region in the closed green line was taken as the breast region. The pixels in the breast region were marked as 1, and those outside the breast region were marked as 0 for binary storage. In this slice, 46 points were manually localized.

### 2.4. GC3D

#### 2.4.1. Image Segmentation

Given an image, a trimap *T* is manually outlined ($T=T_{B}⋃T_{F}⋃T_{U}$). $T_{B}$, $T_{F}$ and $T_{U}$ correspond to the set of pixels in the background, the foreground and the unknown region. The pixel intensity is one of the values $z=\{z_{1},...,z_{i},...,z_{n}\}$. Image segmentation is to group each pixel in $T_{U}$ to $T_{B}$ (α=0) or $T_{F}$ (α=1). Note that α indicates the label of voxels. In GrabCut, α evolves to be either 0 or 1 for each voxel at the end of the iterations, i.e., α={0,1}. A parameter $\underline{\theta }$ describes the grey-level distributions, $\underline{\theta }=\left\{h\left(z;\alpha \right)\right\}$. The task of image segmentation becomes inferring the unknown α for pixels in $T_{U}$ from the learned model $\underline{\theta }$ based on the given data z.

#### 2.4.2. *s*-*t* Graph

**Figure 4:**
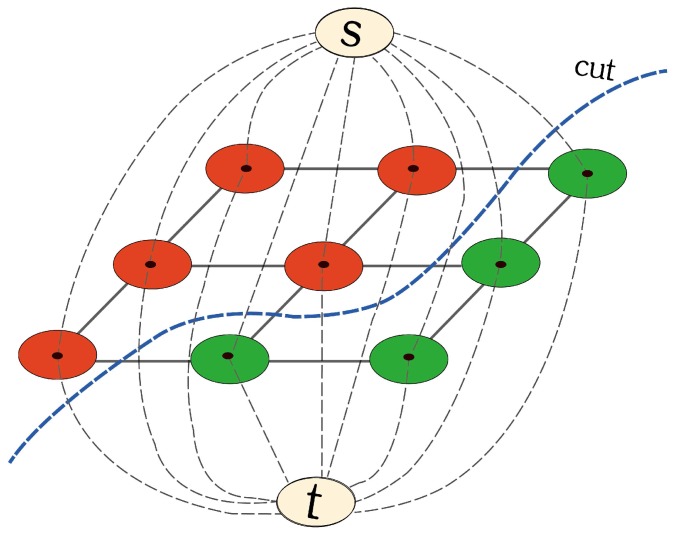
*s*-*t* graph. An image can be expressed as an undirected graph with two additional vertexes (*s* and *t*). Image segmentation is to find a cut (blue dotted line) that separates the foreground (red rounds) from the background (green rounds). The figure can be enlarged to view the details.

An image can be described as an undirected graph, *g* = <*v*, *e*>, where *v* is the vertex or node set and *e* is the edge set [49,50,51]. Two special vertices are added in the graph as shown in Figure 4. One is the source point (*s*), and the other is the sink point (*t*). There are two kinds of edges, solid lines (*N*-links) and dotted lines (*T*-links). The former represents the edges between adjacent ordinary vertices, and the latter indicates the edges between *s* (or *t*) and the ordinary vertices. In addition, the edge is weighted based on the similarity between the vertices on it. Shown in Figure 4, any given pixel is strictly and loosely connected to 4 and 8 neighboring pixels, respectively. One can imagine that in 3D space, there are 6 and 26 neighboring voxels connected to the central one.

#### 2.4.3. Segmentation by Iterative Energy Minimization

GrabCut fulfills image segmentation by energy minimization. The energy function E is defined in Equation 1, where $U(\alpha ,k,\underline{\theta },z)$ concerns the weights in *N*-links (the region energy) and V(α,z) concerns the weights in *T*-links (the boundary energy). The vector $k=\{k_{1},...,k_{l},...,k_{N}\}$ distinguishes GrabCut from other graph cuts methods in the optimization framework, since GrabCut makes use of Gaussian mixture models (GMMs) to model the background and the foreground pixles. GMM is a probabilistic model. It assumes all the data points are obtained from a mixture of a finite number of Gaussian distributions with unknown parameters. To each GMM in the GrabCut method, it contains *K* components, and each pixel is assigned a unique component ($k_{l}\in \left\{1,...,K\right\}$).

(1) $$E\left(\alpha ,k,\underline{\theta },z\right)=U\left(\alpha ,k,\underline{\theta },z\right)+V\left(\alpha ,z\right),$$

GrabCut consists of incomplete initialization and iterative energy minimization. The required user interaction is to draw a rectangle. The region outside and inside the rectangle corresponds to $T_{B}$ and $T_{U}$. Then, the background and the foreground GMM components are preliminarily initialized with pixels in $T_{B}$ (α=0) and in $T_{U}$ (α=1), respectively. Next, a *s*-*t* graph is built, and a coarse segmentation is obtained by using the max-flow min-cut algorithm [52,53]. With updated $T_{B}$ and $T_{F}$, new GMMs are learned, and a new graph is created; thereby, an intermediate segmentation is achieved. Iteratively, GrabCut runs until the energy function E converges. If the segmentation result is unsatisfactory, further user editing can be utilized as a remedy for image segmentation.

#### 2.4.4. Implementation of GC3D

Instead of drawing a rectangle, GC3D generates a polygon for algorithm initialization. It requires the points to be localized between the ring transducer and the breast boundary in the coronal slice nearest to the chest wall. If the polygon covers the whole potential breast region, the initialization is correct. The polygon is then duplicated and propagated to the other slices to save time and energy. At last, a volume mask is created for incomplete labeling.

For medical image modeling, such as in GC3D, each component ($\underline{\theta }$) contains three parameters, one weight (π), one mean (μ) and one covariance (δ). How to calculate the boundary energy $U(\alpha ,k,\underline{\theta },z)$ is shown as follows:

(2) $$U\left(\alpha ,k,\underline{\theta },z\right)=\sum_{n}D\left(\alpha_{n},k_{n},\underline{\theta },z_{n}\right)=\sum_{n}\left\{-logp\left(z_{n}|\alpha_{n},k_{n},\underline{\theta }\right)-log\pi \left(\alpha_{n},k_{n}\right)\right\},$$

where p() is a Gaussian probability distribution and π() are a mixture of weight coefficients. Here we define *T*-links between nodes and *s* are noted as $T_{s}$, and *T*-links between each node and *t* are as $T_{t}$. If one pixel belongs to $T_{F}$, $T_{s}=W$ and $T_{t}=0$, otherwise $T_{s}=0$ and $T_{t}=W$. Note that *W* is the largest, constant edge weight in the *s*-*t* graph. However, if one pixel *x* is in $T_{U}$, its probability is formulated in Equation 3 where the symbol ${}^{\prime }$ denotes the transpose operation.

(3) $$D\left(x\right)=-log\sum_{i=1}^{K}\left\{\frac{\pi (\alpha_{x},i)}{\sqrt{det(\delta \left(\alpha_{x},i\right))}}\times exp\left(\frac{1}{2}{\left(z_{x}-\mu \left(\alpha_{x},i\right)\right)}^{{}^{\prime }}\delta {\left(\alpha_{x},i\right)}^{-1}\left[z_{x}-\mu \left(\alpha_{x},i\right)\right]\right)\right\}.$$

The calculation of the region energy V(α,z) is related to *N*-links, and the major difference comes from how to weigh the edges between these connected nodes in the graph in 3D space. Assuming N(x,y) is the edge weight between any connected nodes of *x* and *y*, V(α,z) is computed as follows,

(4) $$V\left(\alpha ,z\right)=\sum_{(x,y)\in e}\left[\alpha_{x}\neq \alpha_{y}\right]N\left(x,y\right),$$

and the inequality operator (≠) indicates that it yields 0 when $\alpha_{x}$ and $\alpha_{y}$ are equal and 1 if otherwise. Defining Euclidean distance as d(x,y), the edge weight N(x,y) is

(5) $$N\left(x,y\right)\times d\left(x,y\right)=w\times e^{-\beta ||I_{x}-I_{y}\left|\right|}$$

in which $\left|\right|I_{x}-I_{y}\left|\right|$ represents the intensity difference and *w* is a constant. In GC3D, 6-connected voxels are considered, and d(x,y)=1. As for β, it is defined as $\frac{1}{\beta }=\frac{2}{P}\sum_{i=1}^{P}\sum_{j=1}^{Q}\left|\right|I_{i}-I_{j}{\left|\right|}^{2}$, where *P* is the voxel number in the volume and *Q* indicates the number of the connected neighbors to the central voxel.

In summary, two parameters are tunable. One is *K*, the number of Gaussian components in each GMM; and the other is *w*, the constant weighting value in Equation (5). Off-line experiments were carried out to find the optimal *K* (K=2,3,...,9) and *w* (w=30,40,...,90). The results indicated that GC3D is consistent regarding to the change of the tunable parameters (±1% changes in MO). Therefore, we use the suggested values, i.e., K=5 [42] and w=50 [43] in this study.

### 2.5. Experiment Design

The reliability of gold standard data is estimated with the intra- and the inter-observer variability analysis. Intra-observer analysis is based on two sessions of outlining the breast regions by a senior physician (15-year work experience), while inter-observer comparison is between the senior physician and a junior physician (2-year work experience). Three months later, the re-test was performed, following the same test procedure in the benchmark building. Note that the first and the second session from the senior physician is marked as S and $S^{\prime }$, and the session from the junior physician is marked as J.

Four semi-automatic methods were evaluated. Besides GC3D, two other implementations of GrabCut are involved: GC with monochrome slices as its input, and GC3D_26 taking 26-connected neighboring voxels into the computation. In addition, sSNAKE was compared which allows for control point localization [46]. Different from the original method, control points were placed near but not on the breast boundary before contour propagation.

Two more experiments were further conducted. One was to investigate whether localizing points to form a polygon is more user-friendly than drawing a rectangle in the algorithm initialization. The other was to study the robustness when different users (two non-physicians and a junior physician) manipulate GC3D.

### 2.6. Performance Evaluation

To evaluate the reliability of gold standard data, Dice similarity index was used [54]. It is defined as below,

(6) $$DICE=2\frac{\left|X\cap Y\right|}{\left|X|+|Y\right|},$$

where *X* and *Y* are segmentation results, and |·| denotes the number of elements in one volume.

Algorithm performance was estimated from two volume overlap agreement measures and two overlap error measures. The former two measures are target overlap (TO) and mean overlap (MO), and the latter two are false positive (FP) and false negative (FN) errors [55]. Given the segmentation result (*S*) and the ground truth (*G*), TO, MO, FP and FN are correspondingly defined as below,

(7) $$TO=\frac{\left|G\cap S\right|}{\left|G\right|},$$

(8) $$MO=2\frac{\left|G\cap S\right|}{\left|G|+|S\right|},$$

(9) $$FP=\frac{\left|S|-|G\cap S\right|}{\left|G\right|},$$

(10) $$FN=\frac{\left|G|-|G\cap S\right|}{\left|G\right|}.$$

The average time consumption (TC) for each volume was also concerned. It accounts for the time spent on the manual initialization to the end of whole volume segmentation. Note that the user was aware of the time consumption evaluation when performing the task. In Equation 11, $tc_{i}$ stands for the time cost for the *i*th volume, and n=30.

(11) $$TC=\frac{1}{n}\sum_{i=1}^{n}tc_{i}.$$

### 2.7. Software Platform

Three variations of the GrabCut method (GC, GC3D and GC3D_26) were implemented with C++ (Visual Studio 2010, https://www.visualstudio.com/). The computer was equipped with 8 Intel (R) Cores (TM) of 3.70 GHz and 8 GB DDR RAM. The algorithm implementation did not use any optimizations or strategies for algorithm acceleration. Other involved software include OpenCV (http://opencv.org/) and ITK ((http://www.itk.org/). In addition, sSNAKE [46] was utilized with MATLAB (MathWorks, Natick, MA, USA).

To evaluate the reliability of ground truth building, intra-class correlation (ICC) analysis was employed. Two-way mixed effects model (ICC3,1) was used to assess the intra-observer test-retest reliability, and two-way random-effects model (ICC2, k) was used to quantify the inter-observer reliability [56,57]. ICC coefficient values (*r*) ranging from 0.81 to 1.00 indicate excellent reliability; 0.61 to 0.80 good reliability; 0.41 to 0.60 moderate reliability; 0.21 to 0.40 fair reliability and below 0.2 poor reliability [58]. The paired sample *t* test was used to compare the differences in measurements between observers and a significance level of 0.05 was set. Statistical analysis was performed using the R software, Version 3.0.1 (http://www.R-project.org).

## 3. Results

### 3.1. Reliability of the Ground Truth Data

The segmentation results are in good overlap and DICE values are generally greater than 0.96. Furthermore, excellent intra- and inter-observer reliability are observed and ICC coefficient values are all greater than 0.95. In particular, the paired sample *t* test indicates that no significant difference is found between two sessions of breast segmentation by the senior physician (p=0.4359) and between the segmentation results from the senior and the junior physicians (p≥0.3934) in Table 1.

### 3.2. Perceived Evaluation

**Figure 5:**
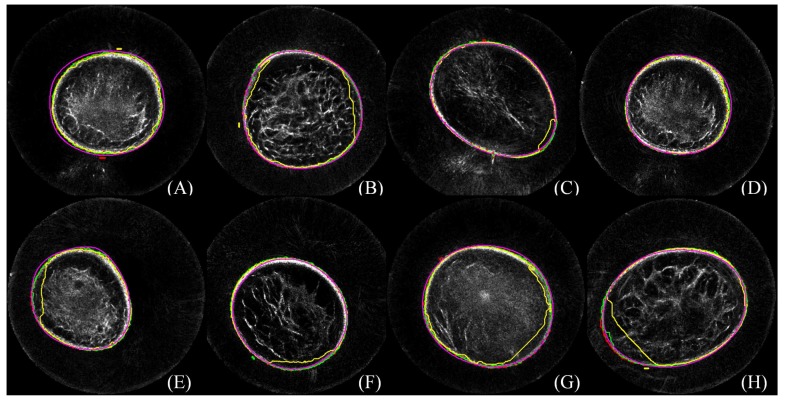
Perceived evaluation of breast segmentation based on eight cases. In each case, lines in red, green, blue, yellow and pink color correspond to segmentation results from manual delineation, GrabCut (GC), Three-dimensional GrabCut (GC3D), GC3D_26 and simplified SNAKE (sSNAKE), respectively. The figure can be enlarged to view the details.

The perceived evaluation of eight cases is demonstrated in Figure 5. In each case, lines in red, green, blue, yellow and pink color correspond to segmentation results from manual delineation, GC, GC3D, GC3D_26 and sSNAKE, respectively. It shows that GC, GC3D and sSNAKE successfully separate the breast from the water region, while GC3D_26 causes over-segmentation. By enlarging the figure, we find that the outlines from sSNAKE are slightly outside of the breast boundary (A, E), while GC and GC3D result in tight outlines.

**Figure 6:**
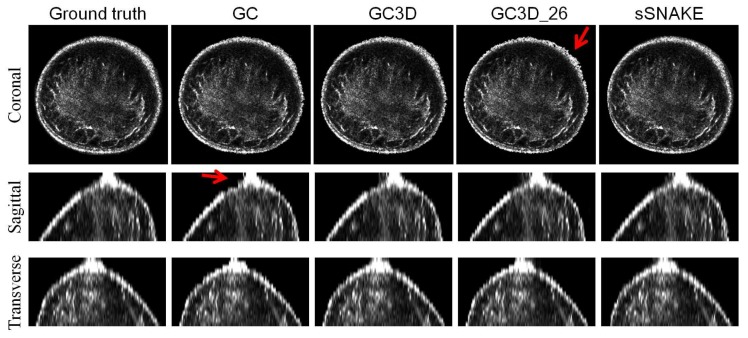
Perceived evaluation of a case from three views. Algorithms successfully separate the breast region from the background. Minor defects are indicated with red arrows; GC3D_26 generates the least smooth breast boundary, and GC leads to jagged edges at the nipple region. Images are interpolated in the sagittal and coronal view and cropped in three views for display purposes. The figure can be enlarged to view the details.

Figure 6 ustrates a case from three views. It shows that algorithms successfully separate the breast from the water region and no major difference is observed among algorithms. However, minor differences can be found. As indicated by the red arrow, GC3D_26 generates the least smooth breast boundary as seen in the transverse view, and GC leads to jagged edges at the nipple region in the coronal view.

### 3.3. Quantitative Comparison

The quantitative comparison is shown in Table 2. It was found that sSNAKE achieved the top accuracy (TO and MO), followed by GC3D and GC, while it also had the highest FN value. On the other hand, GC3D is the most convenient, because it requires the least number of points (≈12 points per volume) to be placed for algorithm initialization. Compared to GC3D, GC, sSNAKE and benchmark building takes 8, 19 and 35 times more points to a volumetric image, respectively. In addition, TC values show that GC3D could process a UST volume within about 1.2 min, followed by GC3D_26 and GC.

### 3.4. Ease-Of-Use

**Figure 7:**
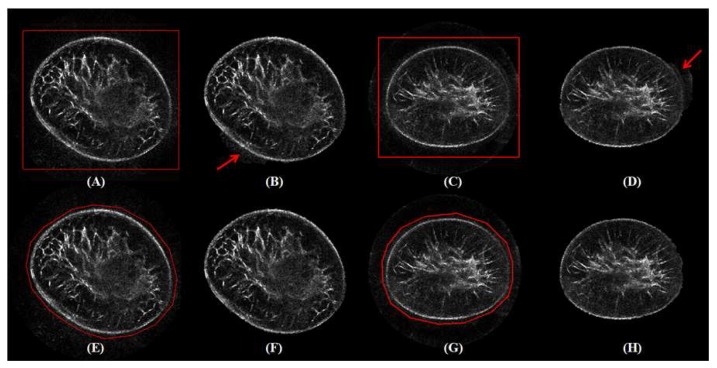
Algorithm initialization of GC3D by drawing a rectangle versus localizing points to form a polygon. Two cases are illustrated from the coronal view and red arrows point to over-segmented regions. The figure can be enlarged to view the details.

Figure 7 shows coronal images with different inputs (A, C, E, G) and corresponding results of the segmented breast regions (B, D, F, H). Note that only the slice with the maximum breast region is illustrated. The red arrows in (B, D) point to the regions with incorrect segmentation. It indicates that polygons lead to better segmentation (F, H) compared to results with rectangles as the input (B, D), respectively.

Performance estimation of GC3D in breast segmentation with different initialization approaches is shown in Table 3. In comparison to the polygon input, a rectangle input results in lower accuracy (TO and MO) and higher error (FN and FP). It requires 3–4 tries to draw an appropriate rectangle and thereby prolongs the time (≈ 50% increase). Note that an appropriate rectangle requires the rectangle covering the breast region while not interacting with the ring transducer. Due to several attempts at selecting an appropriate rectangle, the segmentation time increases. However, when the rectangle contains voxels on the ring transducer, GMMs would be misleading, and thereby, the performance in UST segmentation decreases.

### 3.5. Robustness

**Figure 8:**
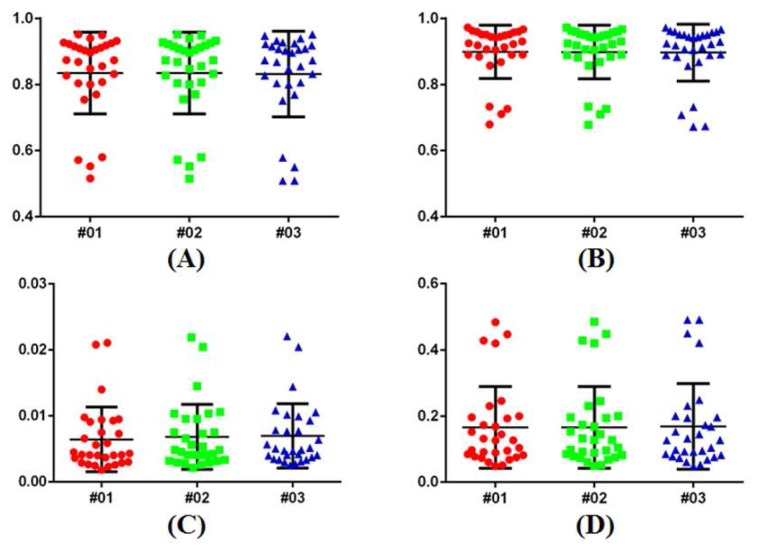
Robustness analysis of GC3D manipulated by different users on UST image segmentation. (**A**) TO; (**B**) MO; (**C**) FP; (**D**) FN. No significant difference is found among observers in each metric.

The robustness analysis is carried out by manipulating GC3D and points are localized for algorithm initialization. Quantitative results from three users (two non-physicians and a physician) are demonstrated in Figure 8. It indicates that the metric values are very close to each other (#01, non-physician: TO = 0.85, MO = 0.91, FP = 0.006, and FN = 0.154; #02, non-physician: TO = 0.84, MO = 0.91, FP = 0.007, and FN = 0.155; and #03, physician: TO = 0.85, MO = 0.91, FP = 0.007, and FN = 0.155). One-way ANOVA indicates no significant difference among each metric (*p*-value > 0.99). The ICC coefficients revealed the measurement agreement among users (*r* > 0.98).

### 3.6. Failure Case Analysis

**Figure 9:**
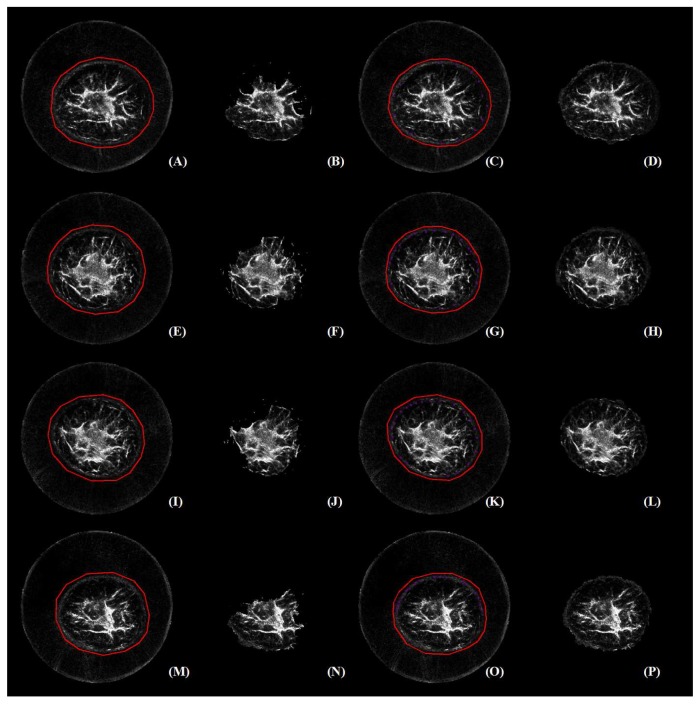
Failure case analysis and additional user editing for image segmentation. It is observed that failed cases share similar image contrast, i.e., black boundary and bright inner tissue. In addition, user editing benefits accurate breast segmentation. The figure can be enlarged to view the details.

Figure 8 reveals that there are four cases in which GC3D failed to segment the major breast regions (TO<0.65). Subsequently, these failure cases were represented and further edited with user interaction. Results before and after user editing are illustrated in Figure 9. The editing was fulfilled with pink dots on the breast boundary, and these voxels were labeled into the foreground ($T_{F}$). Note that the pink dots are visible after the figure is enlarged.

With labels on the breast boundary, the segmentation after editing (D, H, L, P) was more accurate than those before editing (B, F, J, N), respectively. Interestingly, the failure cases (A, E, I, M) shared similar tissue contrast, i.e., black boundary and bright inner tissue in perception. It was also found that with additional labeling, improved breast segmentation was achieved, and major breast regions were separated.

## 4. Discussion

Efficient segmentation of the entire breast in B-mode UST images plays an important role in visualization, analysis and diagnosis of breast disease. This paper explored various implementations of GrabCut (GC, GC3D and GC3D_26) and the simplified SNAKE (sSNAKE) for semi-automatic breast segmentation. Quantitative comparison based on thirty breast volumes indicated that GC3D achieves a good balance of segmentation accuracy, time investment, ease-of-use and robustness. In particular, GC3D only requires users to place several points for algorithm initialization, which improves usability. Therefore, to trade-off between efficiency and accuracy, GC3D is recommended for the breast segmentation task with respect to UST image applications.

The reliability of benchmark building was investigated based on the intra- and the inter-observer variability analysis (Table 1). Statistical analysis showed that there were no significant differences between two sessions of the senior physician and between the session of the senior and the junior physicians. Furthermore, the accuracy of built data sets as gold standard was validated and the segmentation results were in good coincidence (DICE > 0.96). One reason for the excellent correlation in breast segmentation is that the breast regions were outlined by physicians with professional background. The other reason is that two other physicians additionally checked the segmentation results and the benchmark was built with three observers by consensus. It should be noted that the segmentation result of the first session by the senior physician was taken as the gold standard data for further algorithm comparison.

Four semi-automated algorithms were evaluated and GC3D achieved good segmentation within an acceptable time (Table 2). It outperformed GC3D_26. The difference between the algorithms was that GC3D_26 took 20 more connected voxels into consideration. Therefore, we can conclude that additional voxel connections have a negative effect on the image segmentation. Voxels at the corner might induce inaccurate estimation of the region energy V(α,z) (Equation (1)) because of identical d(x,y) (Equation (5)) and anisotropic image resolution. On the other hand, GC3D was better than GC on TO, MO and FN. In GC3D, a *s*-*t* graph was built with voxels in a volume; and in GC, it was based on voxels in a slice. Consequently, GC3D benefits from accurate estimation of GMMs with a large number of sample voxels. It was also observed that GC achieved slightly better FP than GC3D which might be due to the dedicated point localization in each slice when performing GC. Compared to GC3D with one coarse initialization, slice-wise GC refined the localization of points in each slice, and thereby, less background voxels ($T_{B}$) were grouped into the unknown region ($T_{U}$). However, GC required an average of 98 points to be placed for each volumetric image, which increased time and user interaction. If the points were localized once and then propagated to other slices, the time was reduced (1.52 ± 0.46 min), while the segmentation accuracy decreased (TO = 0.79, MO = 0.84, FP = 0.012 and FN = 0.21). It should be noted that GC is easy for deployment on multi-core computers, which will cut down the segmentation time with parallel programming [59,60]. Although sSNAKE suffers from intensive user interaction in addition to complex parameter tuning, it showed promising results in slice-wise breast segmentation in UST images.

It is easy for users to perform GC3D for B-mode UST image segmentation. What a user needs to do is localize several points between the breast boundary and the ring transducer. Compared to GC and sSNAKE, GC3D requires far fewer points to be localized, which is especially beneficial in a setting with large-scale UST image analysis (Table 2). In addition, the point localization is more flexible than drawing a rectangle, regardless of the breast size, shape and position (Table 3). The latter operation increases time cost by 50% because 3–4 attempts are needed in practice. Particularly, drawing a rectangle results in less accurate GMMs, since more voxels in the background ($T_{B}$) are taken into the unknown region ($T_{U}$). B-mode UST images with lower contrast between ambiguous breast boundaries and bright inner tissues (Figure 9) may need additional touch-ups on breast boundaries. Notably, in addition to the ease of use, its robustness to different users is another main advantage of GC3D in clinical applications. In other words, GC3D can be used by non-physicians, which facilitates large-scale image studies (Figure 8). At present, the manual outline of the breast boundary in UST images is accepted, even though it is laborious and tedious. With the help of GC3D, non-physicians may be trusted to outline the breast boundary after distorted slices are removed, and thereby, save time and energy.

Several issues were identified and the most concerned issues were from image preprocessing and benchmark building. First, removing distorted images reduces the challenges in B-mode UST image segmentation. It should be recognized that removing the distorted images is necessary (Figure 1). It is known to us that artifact removal is extremely difficult in medical imaging [61], and the most fundamental solution comes from the upgrading of imaging devices [62,63]. Therefore, in this paper, we followed previous studies [9,12,13] to define the starting and the ending slice. After distorted images were removed, the image contrast is enhanced, and a certain degree of artifacts and noise become invisible in the background region. Note that these artifacts and noise were tackled by the implemented GC3D. Following the selection of the starting and ending slice, an average breast of about 32 mm remained. Since all slices provide useful information for tissue analysis and disease diagnosis, these distorted slices were then processed manually. This made the reproducibility of the selection of the starting and ending slice not as critical.

A few of the other concerns were technical issues. Above all, it should be recognized that a matting tool would likely perform as well as GrabCut in this task. These tools are not limited to graph cut methods [41,42,43] and active contours [26,32,33]; but also for level sets [40], multilevel thresholding [64] and neural networks [65,66]. However, at the preliminary stage of B-mode UST image segmentation, it is helpful to implement a user-friendly tool to facilitate clinical applications, and consequently, this study fills the gap in existing research. This study investigated three variations of GrabCut, since GrabCut has shown superiority among various matting methods [42], and their performance was validated in the task of reflection UST image segmentation. Furthermore, in order to establish the robustness and the speed advantage of GC3D, it is quite meaningful to compare the performance between GC3D and other matting methods [67], such as 3D active contours. However, a previous study [25] indicated that the success of the active contour for attenuation mode UST image segmentation is prone to the suitability of the initialization shape. Thus, a simplified active contour (sSNAKE) [46] was evaluated. It resulted in accurate segmentation, while intensive user interaction hampered its wide application. Moreover, only a handful of literatures are related to UST image segmentation [9,25,27,31]. Besides aforementioned challenges, one probable reason is that UST images are not publicly available, since UST imaging is an emerging modality. In such a situation, the major progress of UST image analysis is made by Delphinus Medical Technologies, Inc. [68] and the team from Karlsruhe Institute of Technology [69]. The manual segmentation of UST images has been currently preferred, particularly because the imaging system is being upgraded. Thus, only a few of articles have been published on this topic. Last but not the least, automated image segmentation is always desirable [25,30,31]. In this study, due to the fixed location of the ring transducer in the B-mode UST images, GC3D has the full potential to be automated. UST imaging device, SoftVue${}^{\mathrm{TM}}$, is continually upgraded, and high-quality UST images can be acquired in the near future. At that time, it will be possible to isolate the whole breast in B-mode UST images by using GC3D in a fully-automated manner. By applying fully-automated methods, reproducible results can be obtained, and diagnostic time can be significantly reduced. As a result, radiologists and physicians alike can be released from this kind of tedious and time-consuming work.

## 5. Conclusions

Three-dimensional GrabCut (GC3D) can be utilized for efficient segmentation of the breast in reflection (B-mode) UST volumetric images. This method has many advantages. It achieves good performance in an acceptable time; and it is user friendly and enables the process to be used for large scale studies. It has the potential to be fully automated and thus save time to release physicians and radiologists from manual breast segmentation. Our future work will focus on the automation of GC3D by incorporating the results from this study.

## Figures and Tables

**Table 1:** Reliability of the ground truth data. *p* values are from the paired *t* test of the segmentation results. The ${}^{\left(*\right)}$ indicates the intra-observer correlation, and ${}^{\left(†\right)}$ denotes the inter-observer correlation.

	SS’	SJ	S’J
DICE	0.9762 ± 0.0417	0.9641 ± 0.0432	0.9657 ± 0.0556
*p* value	0.4359	0.3934	0.7649
ICC	0.9517 ${}^{\left(*\right)}$	0.9588 ${}^{\left(†\right)}$	0.9757 ${}^{\left(†\right)}$

**Table 2:** Quantitative comparison of breast segmentation. The ${}^{\left(‡\right)}$ indicates that it is not comparable between GC3D and sSNAKE because of different implementation languages.

	TO	MO	FN	FP	TC (Min)	No. of Points Localized
Benchmark					11.8 ± 4.82	423 ± 87
GC	0.82	0.90	0.005	0.18	2.37 ± 0.84	98 ± 21
GC3D	0.84	0.91	0.006	0.16	1.23 ± 0.62	12 ± 3
GC3D_26	0.65	0.75	0.023	0.35	1.48 ± 0.65	12 ± 3
sSNAKE ${}^{\left(‡\right)}$	0.89	0.93	0.029	0.11	36.8 ± 5.16	226 ± 32

**Table 3:** Comparison of breast segmentation using GC3D with different initialization approaches.

	TO	MO	FN	FP	TC (Min)
Rectangle	0.83	0.88	0.021	0.17	1.85 ± 1.12
Polygon	0.84	0.91	0.006	0.16	1.23 ± 0.62

## Abbreviations

The following abbreviations are used in this manuscript:

UST	Ultrasound tomography
CT	Computerized tomography
MRI	Magnetic resonance imaging
GC3D	Three-dimensional GrabCut that takes six-connected neighboring voxels into computing
GC	GrabCut
GC3D_26	Three-dimensional GrabCut that takes 26-connected neighboring voxels into computing
sSNAKE	A simplified SNAKE or active contour method
GMM	Gaussian mixture model
TO	Target overlap
MO	Mean overlap
FP	False positive
FN	False negative
TC	Time consumption
